# Enhancing patient-doctor-computer communication in primary care: towards measurement construction

**DOI:** 10.1186/2045-4015-4-4

**Published:** 2015-02-23

**Authors:** Shiri Assis-Hassid, Iris Reychav, Tsipi Heart, Joseph S Pliskin, Shmuel Reis

**Affiliations:** Department of Industrial Engineering and Management, Ben-Gurion University of the Negev, Beer-Sheva, Israel; Department of Business Administration, Ono Academic College, Kiryat Ono, Israel; Department of Industrial Engineering and Management, Ariel University, Ariel, Israel; Department of Health Systems Management, Ben-Gurion University of the Negev, Beer-Sheva, Israel; Faculty of Medicine in the Galilee, Bar Ilan University, Safed, Israel

**Keywords:** Patient-doctor-computer communication, Primary care, Electronic medical record, Communication skills

## Abstract

**Objective:**

The traditional dyadic dynamics of the medical encounter has been altered into a triadic relationship by introducing the computer into the examination room. This study defines Patient-Doctor-Computer Communication (PDCC) as a new construct and provides an initial validation process of an instrument for assessing PDCC in the computerized exam room: the e-SEGUE.

**Material and methods:**

Based on the existing literature, a new construct, PDCC, is defined as *the physician’s ability to provide patient-centered care while using the computer during the medical encounter*. This study elucidates 27 PDCC-related behaviors from the relevant literature and state of the art models of PDCC. These were embedded in the SEGUE communication assessment framework to form the e-SEGUE, a communication skills assessment tool that integrates computer-related communication skills. Based on Mackenzie et al.’s methodological approach of measurement construction, we conducted a two-phased content validity analysis by a general and expert panels of the PDCC behaviors represented in the e-SEGUE. This study was carried out in an environment where EMR use is universal and fully integrated in the physicians’ workflow.

**Results:**

The panels consisted of medical students, residents, primary care physicians, healthcare leaders and faculty of medicine members, who rated and provided input regarding the 27 behaviors. Overall, results show high level of agreement with 23 PDCC-related behaviors.

**Conclusion:**

The PDCC instrument developed in this study, the e-SEGUE, fared well in a rigorous, albeit initial, validation process has a unique potential for training and enhancing patient-doctor communication (PDC) in the computerized examination room pending further development.

## Background

### Patient-doctor communication (PDC) and patient-centered care

Patient-centered care mainly focuses on organizing healthcare delivery around the patient’s needs [1] and is frequently postulated as a preferred approach to patient care [2]. The concept of patient centeredness has received numerous definitions. For example: Balint [3] defines patient-centered medicine as understanding the patient as a unique human being; McWhinney [4] refers to understanding the patient’s experience of illness. Other definitions offer specific components such as: maintaining a bio-psychosocial perspective, understanding the patient as a person, sharing power and responsibility, developing a therapeutic alliance and being aware of the subjectivity of the physician as a person [5–7]. There is no doubt, however, that medical care requires effective physician-patient communication that can be achieved through patient centeredness [6].

The importance of PDC is recognized both in practice and in the literature: In practice, organizations such as the Institute of Medicine (IOM), the Accreditation Council for Medical Education (ACGME) and the American Board of Internal Medicine, require medical students and residents as of 2004 to demonstrate communication competencies in order to receive their certification [8, 9]. In the theoretical field, medical research has attempted to define the necessary ingredients for conducting an effective consultation by establishing guidelines related to physicians’ tasks, strategies and skills that should be carried out during the medical encounter. Guidelines are provided by multiple sources with the most salient ones being : the task approach [10], the three function model [11], the four habits model [12], the Smith model [13], the Kalamazoo consensus statements [14], the Calgary-Cambridge guides [15], the SEGUE framework [16], the Macy Model Checklist [17], the MAAS-Global [18] and the Roter Interaction Analysis System [19].

### PDC in the computerized exam room

The use of computers in healthcare has significantly evolved in the past two decades from word processing, office management, and billing to more complex dimensions of healthcare, such as: diagnosis, consultation, education, and treatment [20]. As computers become an integral part of medical care, the use of Electronic Medical Records (EMRs) in primary care is increasing rapidly. Nearly 100% of Australian physicians use EMRs during the medical encounter. Similar rates have been reported in the Netherlands, New Zealand, Israel and Germany, whereas Canada and the US are falling behind, with implementation rates of 25% [21].

The introduction of EMRs into all levels of care, clearly entails substantial potential advantages to healthcare [20, 22–24]. Therefore, research examining the effects of EMR use on the patient-doctor relationship and patient satisfaction is called for. The computer has been defined as a ‘third party’ in the medical encounter, and has been found to alter the interaction dynamics between physician and patient [25, 26]. It is also clear that the computer has changed physicians’ behavioral patterns during the consultation, thus resulting in a diverting effect on various aspects of patient-centered care [14, 25, 27, 28]. As the computer adds a new dimension to the patient-doctor relationship, it appears necessary to define a new construct that takes into account computer-related communication skills: Patient-Doctor-Computer Communication (PDCC). In order to measure the PDCC construct, we developed a new tool for assessing physician communication skills during the medical encounter while using the computer.

## Objective

This study focuses on defining and validating a measurement tool to assess the necessary communication skills required for establishing effective PDCC which can be viewed as a potential extension of the SEGUE and represented as the e-SEGUE.

## Methods

### Patient-doctor-computer communication (PDCC) – scale development methodology

This study’s primary objective is to develop and validate a physician communication skills assessment tool that is suitable for a computerized work environment, since to date such a tool has not been identified. To this end, we have selected the methodology presented by MacKenzie et al. [29]. It offers specific and structured steps for measurement construction and validation and has been published in Management Information Systems Quarterly (MISQ), which is considered one of the most prestigious journals in the information systems discipline. By applying this structured methodology we wish to carry out a successful and accepted construct and measure. Concerns have been raised that existing literature does not use sufficient validation techniques when developing new instruments [29–31]. MacKenzie et al. [29] attempt to address these concerns and identify the specific shortcomings in existing scale development studies. They assert that these fail to adequately define the construct domain, fail to correctly specify the measurement model and underutilize techniques for establishing construct validity. They offer an updated set of recommendations for scale development that comprise several steps and include: (1) developing a conceptual definition of the construct; (2) generating items that represent the construct and (3) assessing the content validity of the items.

### The e-SEGUE assessment tool development process

#### Step 1: construct conceptualization

Understanding the actual effects of computerization and EMR use on the patient-doctor relationship has not reached its full extent [23, 32]. The literature on PDC in the computerized examination room can be divided into two general categories: descriptive research, which mainly describes physician behavioral patterns during computer use [33] and prescriptive research, which attempts to elicit proper behavioral guidelines for communicating with patients in the computerized exam room [23, 25, 34].

MacKenzie et al. [29] stress the importance of defining the conceptual domain to which the focal construct belongs and the entity to which it applies. In the case before us, the property of the PDCC construct refers to communication skills. The entity to which the construct applies is the physician. In other words, the construct PDCC refers to the physician’s communication skills that are applied to maintain patient-centered care while using the computer in the primary care clinic.

According to the first step of Mackenzie’s methodology, we describe the construct’s conceptual theme, referring to its fundamental necessary and sufficient characteristics. Following this methodology we define a new construct, patient-doctor-computer communication (PDCC) as *the physician’s ability to provide patient-centered care while using the computer during the medical encounter*. We stress, in line with previous research, that the computer must be practically recognized as a third and significant actor in the medical encounter, and thus, relevant communication skills must be acquired and practiced [25, 26].

#### Step 2: item generation

The second step of measurement construction suggested by Mackenzie et al. [29] concentrates on generating the items which will constitute the PDCC assessment tool and will be applied to assess PDCC later on.

The items constituting the PDCC assessment tool have been obtained through a comprehensive literature review which included the state of the art model of evidence-based computer-related communication skills developed by Duke et al. [35]. Overall, we have been able to generate 27 computer-related communication skills that intend to facilitate a patient-centered encounter in the computerized exam room. These were refined and modified as part of our content analysis validation process and resulted in a total of 23 final items.

#### PDCC communication assessment tool – framework selection

In line with a patient-oriented workflow which focuses on details of communication sequences and activities of the medical encounter [1], this study applies the SEGUE framework, which is considered a valid, reliable and acceptable tool for assessing PDC [36]. The choice of the SEGUE is based on a profound evaluation of existing communication skills assessment framework and tools conducted by Schirmer et al. [37]. The evaluation process examined three characteristics: psychometric properties (presence and strength of psychometric data), practicality/usability (rating ease of use when considering who the raters of the instrument would be, complexity of the instrument and length) and overall value (summary of the study’s evaluators’ general impression of the instrument).

Schirmer et al. show that checklists (as applied for example by the SEGUE, Macy Model and Kalamazoo consensus statements) provide clear behavioral definitions for less experienced observers which may improve reliability of the instrument’s rating results. Rating methods which apply criteria scales require a high level of expertise in medical communication in order to provide reliable rating results. None of the existing physician communication assessment tools described in the evaluation process received high evaluations on all parameters (psychometric properties, practicality/usability, and overall value). As such, the researcher is left to make the choice by the best fit for the study’s purpose as well as reasonable psychometric properties. The SEGUE satisfies these two criteria as stated above. The workflow described by the SEGUE views the medical encounter as divided into five main stages (whose initials constitute the acronym): Set the stage, Elicit information, Give information, Understand the patient’s perspective and End the encounter.

#### Step 3: content validity assessment

The third step towards measurement construction according to MacKenzie et al.’s methodology [29] is assessing the content validity of the items. To this end, we have conducted a two-phased experts’ panel. The first phase consisted of an expanded panel, meaning that it targeted a larger pool of participants in relevant fields, such as: faculty members in health administration, residents and interns and medical school students. The second phase included a panel which consisted of a small number of primary care physicians, specifically familiar with the field of patient-doctor communication and implementation of EMRs. The purpose of the first phase was to establish an initial validation of the 27 PDCC behaviors whereas the second phase was targeted towards obtaining the perspective of specialists in the PDCC domain.

Participants were recruited via e-mail invitations.

#### Phase 1 – general panel

The first phase of content validity assessment intended to reach as many participants as possible that have some familiarity with EMRs, patient-doctor communication, patient interviewing and communication skills. The panel took place in several different locations in order to obtain a wide perspective: universities, public hospitals, private hospitals and medical schools. Respondents from faculties of medicine and health administration in Israeli colleges and universities received a personal explanation regarding this study’s purposes and the communication assessment tool at hand. Other respondents who could not be physically reached (practicing physicians and interns), received the questionnaire via e-mail along with a detailed written explanation. Due to difficulties in participant recruitment, the sample should be considered a convenience sample.

Data was collected by distributing paper-based questionnaires, or by using Google’s web survey (Google docs form) which was e-mailed to potential respondents. Responses to all surveys were anonymous. Table 1 illustrates the demographics of the expanded panel participants.

**Table 1:** **Phase 1 – general panel demographics**

Parameter	%
**N**	48
**Gender**	
Female	37.5
Male	54.2
Missing	8.3
**Professional field**	
Primary care	25
Secondary care	2.1
Tertiary care	0
Medical education	12.5
Student	60.4
**Work environment**	
Private clinic	
Public clinic/HMO	25
Private hospital	
Public hospital	
University	12.5
Student	62.5
**Professional experience in years**	
Missing	
0-5	64.6
5-15	14.6
15-25	6.2
25-35	4.2
Over 35	10.4
**Experience in interviewing patients**	
Never interviewed patients	43.7
Interviewed patients a few times	29.2
Years of experience in interviewing patients	27.1
**EMR use**	
Never used an EMR	64.6
Rarely use an EMR	12.5
Use an EMR on a daily basis	22.9

The first survey consisted of 48 respondents that were asked to rate each of the 27 behaviors on a scale ranging from 1 to 5 (where 1 = behavior is not relevant at all for establishing effective physician-patient communication while using the computer, and 5 = behavior is essential for establishing effective physician-patient communication while using the computer). Most of the respondents were students from the Faculty of Medicine in the Galilee, Israel (60.4% of the respondents) who participated in a course addressing physician patient communication in the computerized setting. Other respondents consisted of primary care physicians working in the Israeli public health system (25% of the respondents), as well as university researchers and medical educators from different health sectors (secondary care and medical education) (14.6%).

Percentage of agreement was calculated by adding up the frequencies of high ratings on the scale (4 = behavior is important for measuring the PDCC construct and 5 = behavior is essential for measuring the PDCC construct) for each of the 27 behaviors. We conclude that the rating of 3 is more typically viewed by respondents as a mid-point indicating indifference and, therefore, believe that adding this rating would not adhere to the required rigor we sought to achieve in this test. As such, we have computed only ratings of 4 and 5 in order to decide whether or not the item is essential for measuring the construct (in line with the definition of content validity).

#### Between-groups analysis

Since students accounted for 60% of the sample, we decided it would be appropriate to carry out an analysis of the differences in ratings between students and professional respondents. In order to do so, we split the respondents’ data into two groups: students and professionals. In order to find out whether there was a statistically significant difference in the ratings provided by each group, we carried out a Mann-Whitney’s U test which is suitable since our sample is less than 30 participants in each group, a normal distribution cannot be assumed and ratings were provided on a categorical scale.

#### Phase 2 - experts panel

The purpose of the second panel was to bring together primary care physicians, experts in the field of patient-doctor communication and proficient in PDCC, in order to discuss the first panel’s results and the 27 PDCC behaviors. This phase included 8 participants - leaders in national healthcare decision-making, CEOs of private and public hospitals, the chief of information technology in the public health administration, head of the primary care unit in one of the largest HMOs in Israel and physicians that are responsible for the PDC curriculum in medical schools.

The physicians at hand hold many years of experience in patient interviewing as well as implementing EMRs into the primary care workflow. As such, they are best informants for the task of refining the e-SEGUE items.

The panel members were asked to follow the SEGUE workflow while evaluating the PDCC construct via the measurement items. The expert opinions also revealed agreement regarding the need to rename and reframe the SEGUE to *e-SEGUE* in line with the adoption of the PDCC construct.

## Results

### Phase 1 – general panel

Table 2 illustrates the results of the first stage of the content validity analysis – the general panel. The table presents the level of agreement that the item should be included in order to measure PDCC, while taking into account ratings that view each behavior as important or essential for establishing effective PDCC. A threshold of 50% was set to determine sufficient agreement that the behavior should be maintained. Meaning that behaviors receiving under 50% agreement were considered for further discussion. Items that received low level of agreement (under 50%) are marked with “*”. This stage resulted in a total of 20 items that received high ratings (over 50% of agreement). The seven items that received considerably low ratings were assessed in the second phase – the experts’ panel.

**Table 2:** **Frequencies of item ratings (N=48)**

Item number	Item	Percentage of agreement (%)	Mean	Std. dev.
1.	Arrange the room to allow both patient and physician to see the screen	50	3.63	.841
2.	Preview the EMR before entering or having the patient enter the room	60.4	3.69	1.055
3.	Introduce yourself before turning to the computer	93.7	4.71	.874
4.	Introduce the computer and its role to the patient, while identifying the patient in the EMR	33.3*	3.15	1.010
5.	Begin the encounter with your patient’s concerns	95.9	4.60	.574
6.	Summarize and briefly touch-type the visit’s agenda	77.1	4.13	.866
7.	Do not interrupt the patient while he is talking due to computer guided questions/prompts	83.3	4.33	.753
8.	Establish reason for visit primarily based on the patient’s needs rather than computer prompts	85.5	4.29	.713
9.	Describe the security and confidentiality of the patient’s electronic record information	37.5*	3.06	1.262
10.	Discuss antecedent treatments while browsing the computerized record	48*	3.54	.898
11.	Tell the patient what you are doing as you turn to the computer	81.2	3.98	.729
12.	Reposition the screen so that it is closer to the patient	29.2*	3.15	.945
13.	Point to relevant areas on the screen	54.2	3.56	.920
14.	Signal shifts toward the computer, let the patient know that you are still attending to his or her needs	83.4	4.10	.660
15.	Read back what you have written followed by looking at your patient	60.4	3.71	.922
16.	Use transition statements to the computer, signpost, use real-time typing, read-back	47.9*	3.52	.772
17.	Encourage patient participation in building their charts	31.3*	2.92	1.007
18.	Demonstrate sufficient typing skills	64.6	3.67	.996
19.	Verify patient literacy, primary language, and visual acuity to optimize computer use	41.7*	3.19	1.142
20.	Print out or share: care plans, medication lists, office notes, information, follow up appointments	62.5	3.69	1.133
21.	Discuss medical issues and prevention strategies while using computer resources	54.2	3.54	1.031
22.	Initiate/acknowledge patient requests for on-line information (data, screen sharing)	62.5	3.73	.939
23.	Teach the patient about his own body and situation by providing feedback from tests, diagnosis, showing test results on the screen or print out	70.9	3.94	.954
24.	Use verbal and non-verbal cues: eye gaze, affirmative head nodding while patient is talking	89.6	4.46	.743
25.	While typing on the computer, use verbal skills that demonstrate active listening: continuers (uh-huh, go on, I see), echoing statements (back channeling), short requests (tell me more), and short summarizing statements	85.4	4.21	.683
26.	When the patient is talking or when information is provided to him, face the patient: head, eyes, and torso toward the patient, remove hands from the keyboard or mouse, push the monitor away, and give the patient her undivided attention	77.1	4.21	.798
27.	Provide patient handouts (or Web site references) and information about community support services, medication side effects, and follow-up appointments	60.4	3.75	.978

* = items that received a relatively low percentage of agreement (<50%).

### Between-groups analysis

Most of the respondents (60%) were students from the Faculty of Medicine. Though they have participated in a course addressing physician patient communication in a computerized setting, they had little or no experience with working with EMRs or integrating them into an actual medical encounter. In order to find out whether there are significant differences in the ratings provided by students (which lack experience in patient interviewing and EMR use) and the more professional respondent, we carried out the Mann–Whitney U test.

Mann-Whitney’s U test was carried out for each of the items and was based on splitting the sample into two groups of subjects. When the total sample is smaller than N = 61, SPSS can produce the exact probability.

The student group consists of 29 participants (N = 29) and the professionals group consists of 19 (N = 19). Results of the Mann–Whitney U test were non-significant for all items, meaning that we could not show differences in ratings between the two groups, consequently, it is appropriate to combine the two groups into one sample Table 3 shows the results of the test. It is important to note that these results are restricted to the sample size.

**Table 3:** **Mann Whitney’s U test results**

Item number	Item	Mann-Whitney U	Exact sig. (2-tailed)	Median (group 1)	Std. (group 1)	Median (group 2)	Std. (group 2)
1.	Arrange the room to allow both patient and physician to see the screen	222.000	.220	4.00	.850	3.00	1.433
2.	Preview the EMR before entering or having the patient enter the room	248.000	.562	4.00	1.091	3.00	.597
3.	Introduce yourself before turning to the computer	231.500	.167	5.00	1.088	3.00	.885
4.	Introduce the computer and its role to the patient, while identifying the patient in the EMR	267.500	.865	3.00	1.047	3.00	.841
5.	Begin the encounter with your patient’s concerns	252.000	.583	5.00	.632	4.00	1.017
6.	Summarize and briefly touch-type the visit’s agenda	234.500	.359	4.00	.861	5.00	.229
7.	Do not interrupt the patient while he is talking due to computer guided questions/prompts	211.500	.220	4.00	.774	3.00	.976
8.	Establish reason for visit primarily based on the patient’s needs rather than computer prompts	264.500	.562	4.00	.702	5.00	.478
9.	Describe the security and confidentiality of the patient’s electronic record information	265.000	.167	3.00	1.319	4.00	.882
10.	Discuss antecedent treatments while browsing the computerized record	256.000	.865	3.00	.949	5.00	.697
11.	Tell the patient what you are doing as you turn to the computer	267.000	.583	4.00	.778	4.00	.749
12.	Reposition the screen so that it is closer to the patient	228.000	.359	3.00	.944	3.00	1.202
13.	Point to relevant areas on the screen	263.500	.157	4.00	.907	4.00	.838
14.	Signal shifts toward the computer, let the patient know that you are still attending to his or her needs	235.500	.809	4.00	.711	4.00	.667
15.	Read back what you have written followed by looking at your patient	261.500	.832	4.00	.850	3.00	.946
16.	Use transition statements to the computer, signpost, use real-time typing, read-back	235.000	.680	3.00	.783	4.00	.964
17.	Encourage patient participation in building their charts	267.000	.864	3.00	1.145	4.00	.577
18.	Demonstrate sufficient typing skills	238.500	.295	4.00	1.099	4.00	1.046
19.	Verify patient literacy, primary language, and visual acuity to optimize computer use	249.500	.797	3.00	.967	4.00	.761
20.	Print out or share: care plans, medication lists, office notes, information, follow up appointments	274.000	.341	4.00	1.168	3.00	.780
21.	Discuss medical issues and prevention strategies while using computer resources	242.500	.768	3.00	1.121	4.00	.838
22.	Initiate/acknowledge patient requests for on-line information (data, screen sharing)	243.500	.374	4.00	.891	3.00	1.374
23.	Teach the patient about his own body and situation by providing feedback from tests, diagnosis, showing test results on the screen or print out	260.000	.853	4.00	1.093	4.00	1.108
24.	Use verbal and non-verbal cues: eye gaze, affirmative head nodding while patient is talking	257.500	.422	5.00	.862	4.00	.885
25.	While typing on the computer, use verbal skills that demonstrate active listening: continuers (uh-huh, go on, I see), echoing statements (back channeling), short requests (tell me more), and short summarizing statements	272.500	.576	4.00	.726	4.00	1.032
26.	When the patient is talking or when information is provided to him, face the patient: head, eyes, and torso toward the patient, remove hands from the keyboard or mouse, push the monitor away, and give the patient her undivided attention	253.000	.980	4.00	.759	4.00	.705
27.	Provide patient handouts (or Web site references) and information about community support services, medication side effects, and follow-up appointments	264.000	.484	4.00	1.048	5.00	.507

### Phase 1 – general panel

Table 2 illustrates the results of the first stage of the content validity analysis – the general panel. The table presents the level of agreement that the item should be included in order to measure PDCC, while taking into account ratings that view each behavior as important or essential for establishing effective PDCC. A threshold of 50% was set to determine sufficient agreement that the behavior should be maintained. Meaning that behaviors receiving under 50% agreement were considered for further discussion. Items that received low level of agreement (under 50%) are marked with “*”. This stage resulted in a total of 20 items that received high ratings (over 50% of agreement). The seven items that received considerably low ratings were assessed in the second phase – the experts’ panel.

### Phase 2 – experts panel

Table 4 illustrates the results of the second phase, experts’ panel assessment of the items. More specifically, it presents items that should be maintained/discarded/modified in light of the first, expanded panel and in general.

**Table 4:** **Suggested modifications – experts panel**

Original item number	Item	Suggested modification
2	Preview the EMR before entering or having the patient enter the room	Discard item
4	Introduce the computer and its role to the patient, while identifying the patient in the EMR	Keep item
8	Establish reason for visit primarily based on the patient’s needs rather than computer prompts	Discard – similar to item 7
9	Describe the security and confidentiality of the patient’s electronic record information	Keep and add: if needed
10	Discuss antecedent treatments while browsing the computerized record	Keep item
12	Reposition the screen so that it is closer to the patient	Keep and add: or in view of the patient
14	Signal shifts toward the computer, let the patient know that you are still attending to his or her needs	Discard – similar to item 11
15	Read back what you have written followed by looking at your patient	Modify item into: Read back what you have written and add item: Type-in and document information provided by the patient
16	Use transition statements to the computer, signpost, use real-time typing, read-back	Discard – similar to items 25, 26 (verbal and non-verbal communication skills)
17	Encourage patient participation in building their charts	Keep item: Item is important as it offers the patient the opportunity to correct errors in his record and/or approve certain information in his chart.
19	Verify patient literacy, primary language, and visual acuity to optimize computer use	The issue of patient literacy is extremely important especially while using the computer for patient education purposes
24	Use verbal and non-verbal cues: eye gaze, affirmative head nodding while patient is talking	Discard – does not refer specifically to EMR use and is similar to 25, 26.

#### Items that should be discarded

The expert panels’ results revealed that previewing the EMR before the patient enters the room is, in most cases, not possible and therefore the item should be discarded. The panel agreed that several items should be discarded due to an overlap with other items including: establishing reason for visit based on patient need, signaling shifts towards the computer, using transition statements and using verbal and non-verbal cues regardless of the computer. Note that these items were not regarded as unnecessary for establishing PDCC, as they merely overlapped with other existing items.

#### Items that should be maintained

The experts argued that introducing the computer and its role is an important task and entails involving the computer in the encounter as an actual actor. Moreover, there are medical settings that require identification of the patient and uploading his EMR by using a magnetic card (e.g., in Israel). The action of requesting the card and uploading the EMR may disrupt the flow of the conversation and cause the physician to interrupt the patient, yet is unavoidable. Describing security and confidentiality was regarded by the panel as necessary in specific scenarios. Repositioning the screen in order to share information with the patient was also regarded as important since it can enhance the feeling of collaboration and patient involvement, while allowing the patient to confirm/correct his EMR information. Encouraging patient participation in building their charts also offers the patients an opportunity to correct errors in their records and/or approve certain information in their charts. The issue of patient literacy has been raised by the panel as extremely important, especially while using the computer for patient education purposes and therefore should be included.

#### Items that should be modified

The panel suggested that reading back information is an important task that helps verify information as well as avoid awkward silence. However, the panel members agreed that it may be too difficult to type-in, read back and look at the patient at the same time. Therefore, looking at the patient at this time had been discarded from the items. The panel members suggested a new item for further consideration: *Type-in and document information provided by the patient*, as this basic task may, at many times, be neglected or left to the end of the encounter.

Table 5 presents the final 23 behaviors which constitute effective PDCC based on the final results of the experts’ panel, composing the e-SEGUE measurement tool.

**Table 5:** **PDCC final behaviors**

	**Set the stage**
1	Arrange the room to allow both patient and physician to see the screen
2	Introduce yourself before turning to computer
3	Introduce the computer and its role to the patient, while identifying the patient in the EMR with magnetic card
4	Begin the encounter with your patient’s concerns
5	Type in and verbally summarize the visit’s agenda* agenda = all patient’s medical issues and prioritizing and deciding what will be addressed during the visit
6	Do not interrupt the patient while he is talking due to computer guided questions/prompts
7	Describe the security and confidentiality of the patient’s electronic record information if needed
	**Elicits Information**
8	Discuss antecedent treatments while browsing the computerized record
9	Tell the patient what you are doing as you turn to the computer
10	Reposition the screen so that it is closer to the patient / in view
11	Point to relevant areas on the screen
12	Read back what you have written
13	Involve patient in verifying his EMR data accuracy and completeness
14	Type-in and document information provided by the patient
15	Demonstrate sufficient typing skills
	**Give Information**
16	Verify patient’s literacy, primary language, and visual acuity to optimize computer use
17	Print out or share patient education material from the EMR: care plans, medication lists, office notes, test results
18	Discuss medical issues and prevention strategies while using computer resources
19	Provide computer-based information other than the EMR (including: data, screen sharing) or positively acknowledge on-line information provided by the patient
20	Teach the patient about his own body and situation by providing feedback from tests, diagnosis, showing test results on the screen or print out
	**Understand the patient’s perspective**
21	Apply verbal communication skills while using the computer, use **verbal skills** that demonstrate active listening: continuers (uh-huh, go on, I see), echoing statements (back channeling), short requests (tell me more), and short summarizing statements
22	Apply **non-verbal** communication skills while using the computer: when the patient is talking or when information is provided to him, face the patient: head, eyes, and torso toward the patient, remove hands from the keyboard or mouse, push the monitor away, and give the patient her undivided attention
	**End the encounter**
23	Provide patient handouts (or Web site references) and information about community support services, medication side effects, and follow-up appointments

## Study limitations

The first phase of content validity assessment mainly consisted of students from the Faculty of Medicine (60.4% of the respondents). Though they participated in a course addressing physician patient communication in the computerized setting, they had little or no experience with working with EMRs or integrating them into an actual medical encounter. We recognize that it is of great value that respondents of the content validity stage represent the target population as much as possible. However, it is important to note that this study’s target population is very specific (primary care physicians with sufficient experience in patient interviewing and EMR use) and as such, studies which target this specific population usually use relatively small sample sizes [38, 39]. Mackenzie et al. [29] also recognize that in many cases, reaching raters that are representative of the target population may not always be possible and therefore accept using college educated students as well as raters that have sufficient intellectual ability as well. In this particular case, however, the topic of PDCC is rather familiar to the wider population as well, since most have experienced PDCC from the patient’s perspective. Moreover, we have conducted a statistical test of the differences in ratings between the students group and the rest of the respondents and found that there are no significant differences. The second phase (experts panel) included best informants and as such was lean in terms of participants.

Another limitation is that the first panel’s questionnaires were sent to approximately 200 respondents, yet we have no tracking of the demographics of those who did not participate.

It is also important to note that this study took place in an environment in which EMR use is mandatory and well integrated into primary care physicians’ workflow. This may create a certain bias in ratings of the items. However, since EMR implementation is considerably increasing around the world, we presume that the results are applicable to other settings as well.

Finally, this study has not focused per-se on the patient’s perspective regarding the PDCC behaviors. Such input is extremely valuable. This point as well as further statistical measures for validating the e-SEGUE are called for.

## Discussion

For maximizing the significant potential of EMR systems to positively impact patient care quality and safety, they must be integrated into the medical encounter in a manner that supports patient centerdness [35, 40–42]. The change in PDC dynamics, caused by EMR use [25, 32, 43, 44], requires reexamining the necessary skills and communication behaviors for establishing effective PDC. Since existing communication assessment tools and models do not take computer use into account [13, 19, 35, 45, 46], this study identified 27 behaviors that will bridge this existing gap and can be considered for further examination in the extended framework represented by the e-SEGUE.

The identification of these items was based on a careful literature review, wider students’ perspectives as well as international expert consultation. Subsequently, a validation procedure based on user feedback reduced the number of items suitable for the assessment tool down to 23. The Mackenzie et al.’s methodology for measurement construction was meticulously followed, and the choices made (such as the SEGUE framework selection) carefully defended in the former sections. As far as we can tell, this is the first attempt of its kind to make such an instrument available for service, education and research. As such, it is a ground-breaking study.

The e-SEGUE developed in this study still requires further steps of tool development and validation, such as: assessing the scale’s validity (sensitivity, concurrent validity, inter-rater reliability), developing norms for the scale, piloting the instrument, and additional validity and reliability testing. Moreover, the patient perspective regarding these behaviors as well as an empirical evaluation of patient satisfaction related to enhanced practitioner computer-side skills has not been part of this study. Such input would be quite valuable in the future.

It is also important to note that this study took place in a healthcare system in which EMR use is practically universal and integrated in all physicians’ workflow for many years (up to 23 years in some of the settings [25]). However, since EMR implementation is considerably increasing around the world, we presume that this study’s results are applicable to other settings in which EMRs are still under deployment as well. Moreover, in settings in which EMR systems are being deployed, the e-SEGUE will be a very welcome addition to the training and monitoring of the newly deployed innovation.

## Significance

The proposed construct and instrument are a necessary step in making sure that the new era of PDCC is represented by corresponding basic definitions. They can be applied for teaching, service and research purposes in Healthcare Management and Health Professions Education. It can likewise serve as a benchmarking tool for education and practice. The PDCC behaviors developed and initially validated in this study will contribute to fostering patient-centered medicine in the computerized setting, currently compromised due to the cognitive demands involved in computer use at the clinic as well as lack of awareness and training in the domain [8, 23, 47]. Enhancing patient-centered care at the primary care level and improving the encounter can contribute towards providing better quality of care.

## Conclusion

The PDCC construct, as well as the new instrument, the e-SEGUE, offer promising initial steps in making training and monitoring of PDCC skills in healthcare possible in a valid manner. Pending its further development, it can serve as the benchmarking instrument that will enhance of patient-centered skills in the computerized setting.

## Authors’ information

Shira Assid-Hassid is a PhD student at the Department of Industrial Engineering and Management in Ben-Gurion University of the Negev in Israel. Ms. Assis-Hassid has 5 years of work experience in the field of customer relationship management (CRM) and has also been part of the team evaluating the social aspects of wearable computing in the workplace as part of the EU (FP7) funded project: wearIT@work. Her research is interdisciplinary and draws from the fields of: Organizational Culture, Psychology, Sociology, Information Systems and Healthcare Communication.

Dr. Iris Reychav holds an M.Sc in Industrial Engineering and Management, and has 10 years’ experience at Motorola in the field of information systems. She earned her Ph.D. from Bar-Ilan University’s Graduate School of Business Administration, and did her post- doctoral work at the Faculty of Management of Tel Aviv University in cooperation with INSEAD. Today she is a Senior lecturer at Ariel University. Her current research interests include knowledge management technologies, mobile learning and technologies in health and education, and customer relationships management.

Dr. Tsipi Heart is a senior lecturer at the Faculty of Business Administration, Ono Academic College in Israel. Her research interests are within information systems innovation such as cloud computing and SaaS, and strategic impact of information systems particularly in healthcare. Her work has been published in journals as DATA BASE, JAIS, IJMI, MDM, JITTA, IJEB, JGITM, among others.

Prof. Joseph Pliskin Joseph Pliskin is the Sidney Liswood Professor of Health Care Management at Ben-Gurion University of the Negev in the Departments of Industrial Engineering and Management and Health Systems Management.

He received his BSc degree in Mathematics and Statistics from the Hebrew University in Jerusalem (1969) and SM (1970) and PhD (1974) degrees in Applied Mathematics (Operations Research) from Harvard University. Prof. Pliskin’s research interests focus on clinical decision-making, operations management in health care organizations, cost-benefit and cost-effectiveness analysis in health and medicine, technology assessment, utility theory and decision analysis.

Prof. Shmuel P. Reis, MD, MHPE is a family physician in northern Israel, serving within an integrated health and social services center he pioneered over 37 years ago. He is Professor and head of Faculty Development and Clinical Skills Course Director in the Bar Ilan University Faculty of Medicine in the Galilee (Safed, Israel). He is adjunct Professor at Warren Alpert Medical School of Brown University (Providence, RI. USA). He is also the chairperson for HEALER – Israel Association for medical Education (under construction).

## References

[1] Ozkaynak M, Brennan PF, Hanauer DA, Johnson S, Aarts J, Zheng K, Haque SN (2013). Patient-centered care requires a patient-oriented workflow model. J Am Med Inform Assoc.

[2] Bertakis KD, Azari R (2011). Determinants and outcomes of patient-centered care. Patient Educ Couns.

[3] Balint M (1964). The doctor, his patient and the illness.

[4] McWhinney I (1989). The need for a transformed clinical method. Comm Med Patient.

[5] Mead N, Bower P (2000). Patient-centredness: a conceptual framework and review of the empirical literature. Soc Sci Med.

[6] Mead N, Bower P, Hann M (2002). The impact of general practitioners’ patient-centredness on patients’ post-consultation satisfaction and enablement. Soc Sci Med.

[7] Smith RC, Dwamena FC, Grover M, Coffey J, Frankel RM (2011). Behaviorally defined patient-centered communication—a narrative review of the literature. J Gen Intern Med.

[8] Frankel R, Altschuler A, George S, Kinsman J, Jimison H, Robertson NR, Hsu J (2005). Effects of exam‒room computing on clinician–patient communication. J Gen Intern Med.

[9] Stein T, Frankel RM, Krupat E (2005). Enhancing clinician communication skills in a large healthcare organization: a longitudinal case study. Patient Educ Couns.

[10] Pendleton D, Schofield T, Tate P, Havelock P (1984). The consultation: an approach to learning and teaching: Oxford University Press Oxford.

[11] Bird J, Cohen-Cole SA (1990). The three-function model of the medical interview: an educational device. Adv Psychosom Med.

[12] Frankel RM, Stein T (1999). Getting the most out of the clinical encounter: the four habits model. Perm J.

[13] Smith RC, Marshall-Dorsey AA, Osborn GG, Shebroe V, Lyles JS, Stoffelmayr BE, Van Egeren LF, Mettler J, Maduschke K, Stanley JM (2000). Evidence-based guidelines for teaching patient-centered interviewing. Patient Educ Couns.

[14] Makoul G (2001). Essential elements of communication in medical encounters: the Kalamazoo consensus statement. Acad Med.

[15] Kurtz SM, Silverman JD (1996). The Calgary—Cambridge Referenced Observation Guides: an aid to defining the curriculum and organizing the teaching in communication training programmes. Med Educ.

[16] Makoul G (2001). The SEGUE Framework for teaching and assessing communication skills. Patient Educ Couns.

[17] Kalet A, Pugnaire MP, Cole-Kelly K, Janicik R, Ferrara E, Schwartz MD, Lipkin M, Lazare A (2004). Teaching communication in clinical clerkships: models from the macy initiative in health communications. Acad Med.

[18] van Thiel J, Ram P, van Dalen J (2000). MAAS-global manual.

[19] Roter D, Larson S (2002). The Roter interaction analysis system (RIAS): utility and flexibility for analysis of medical interactions. Patient Educ Couns.

[20] McGrath JM, Arar NH, Pugh JA (2007). The influence of electronic medical record usage on nonverbal communication in the medical interview. Health Informatics J.

[21] Jha AK, Doolan D, Grandt D, Scott T, Bates DW (2008). The use of health information technology in seven nations. Int J Med Inform.

[22] Chaudhry B, Wang J, Wu S, Maglione M, Mojica W, Roth E, Morton SC, Shekelle PG (2006). Systematic review: impact of health information technology on quality, efficiency, and costs of medical care. Ann Intern Med.

[23] Shachak A, Reis S (2009). The impact of electronic medical records on patient–doctor communication during consultation: a narrative literature review. J Eval Clin Pract.

[24] Lau F, Price M, Boyd J, Partridge C, Bell H, Raworth R (2012). Impact of electronic medical record on physician practice in office settings: a systematic review. BMC Med Inform Decis Mak.

[25] Margalit RS, Roter D, Dunevant MA, Larson S, Reis S (2006). Electronic medical record use and physician-patient communication: an observational study of Israeli primary care encounters. Patient Educ Couns.

[26] Pearce C, Dwan K, Arnold M, Phillips C, Trumble S (2009). Doctor, patient and computer–A framework for the new consultation. Int J Med Inf.

[27] Patel VL, Arocha JF, Kushniruk AW (2002). Patients’ and physicians’ understanding of health and biomedical concepts: relationship to the design of EMR systems. J Biomed Inform.

[28] Booth N, Robinson P, Kohannejad J (2004). Identification of high-quality consultation practice in primary care: the effects of computer use on doctor-patient rapport. Inform Prim Care.

[29] MacKenzie SB, Podsakoff PM, Podsakoff NP (2011). Construct measurement and validation procedures in MIS and behavioral research: Integrating new and existing techniques. MIS Quarterly.

[30] Straub DW (1989). Validating instruments in MIS research. MIS Quarterly.

[31] Boudreau M, Gefen D, Straub DW (2001). Validation in information systems research: A state-of-the-art assessment. MIS Quarterly.

[32] Pearce C, Arnold M, Phillips C, Trumble S, Dwan K (2011). The patient and the computer in the primary care consultation. J Am Med Inform Assoc.

[33] Harzmark G, Brownbridge G, Fitter M, Evans A (1984). Consultation use of a computer by general practitioners. J R Coll Gen Pract.

[34] Ventres W, Kooienga S, Vuckovic N, Marlin R, Nygren P, Stewart V (2006). Physicians, patients, and the electronic health record: an ethnographic analysis. Ann Fam Med.

[35] Duke P, Frankel RM, Reis S (2013). How to integrate the electronic health record and patient-centered communication into the medical visit: A skills-based approach. Teach Learn Med.

[36] Skillings JL, Porcerelli JH, Markova T (2010). Contextualizing SEGUE: evaluating residents’ communication skills within the framework of a structured medical interview. J Grad Med Educ.

[37] Schirmer JM, Mauksch L, Lang F, Marvel MK, Zoppi K, Epstein RM, Brock D, Pryzbylski M (2005). Assessing communication competence: a review of current tools. Fam Med.

[38] Vishwanath A, Singh SR, Winkelstein P (2010). The impact of electronic medical record systems on outpatient workflows: a longitudinal evaluation of its workflow effects. Int J Med Inf.

[39] Poissant L, Pereira J, Tamblyn R, Kawasumi Y (2005). The impact of electronic health records on time efficiency of physicians and nurses: a systematic review. J Am Med Inform Assoc.

[40] Hartzband P, Groopman J (2008). Off the record--avoiding the pitfalls of going electronic. N Engl J Med.

[41] Blumenthal D, Glaser JP (2007). Information technology comes to medicine. N Engl J Med.

[42] Fiks AG, Alessandrini EA, Forrest CB, Khan S, Localio AR, Gerber A (2011). Electronic medical record use in pediatric primary care. J Am Med Inform Assoc.

[43] Shachak A, Reis S, Pearce C (2013). Patient-physician interactions and electronic health records. JAMA.

[44] Pearce C, Arnold M, Phillips CB, Trumble S, Dwan K (2012). The many faces of the computer: An analysis of clinical software in the primary care consultation. Int J Med Inf.

[45] Smith RC (2002). Patient-centered interviewing: an evidence-based method: Lippincott Williams & Wilkins.

[46] Kurtz SM, Silverman J, Draper J (2005). Teaching and learning communication skills in medicine: Radcliffe Pub.

[47] Ventres W, Kooienga S, Marlin R (2006). EHRs in the exam room: tips on patient-centered care. Fam Pract Manag.

